# A Mixed‐Methods Study Exploring How Food Insecurity Screening can be Embedded in Routine Mental Health Care for Adults With Severe Mental Illness

**DOI:** 10.1111/jhn.70257

**Published:** 2026-04-27

**Authors:** Emma Tuschick, Emma Giles, Jo Smith

**Affiliations:** ^1^ School of Health and Life Sciences Teesside University Middlesbrough UK

**Keywords:** co‐production, food insecurity, mental healthcare, public health, screening, severe mental illness

## Abstract

**Background:**

Food insecurity (FI) is a pressing issue for adults with Severe Mental Illness (SMI), with evidence showing a higher prevalence in this group compared to the general population. However, routine screening for FI in adults with SMI within healthcare settings is currently lacking. Research has indicated that lack of access to adequate food may affect medication adherence and overall health outcomes in this population. This study explored how FI screening can be integrated into routine healthcare practices, co‐produced with individuals with lived experience of SMI, in order to improve early identification, enhance acceptability, and support implementation within mental health services.

**Methods:**

A mixed‐methods exploratory study was conducted between April and June 2025 within one NHS mental health Trust in North East England. Semi‐structured interviews were undertaken with peer‐support workers with lived experience of SMI (*n* = 4), and an anonymous online survey was completed by mental health professionals across disciplines (*n* = 40). Interview data were analysed thematically, and survey data were analysed using descriptive statistics and content analysis of open‐text responses.

**Results:**

Peer‐support workers identified missed opportunities in healthcare where FI could have been addressed, and expressed preferences for flexible, non‐stigmatising screening methods. Barriers included time constraints, stigma, and lack of training, while solutions focused on embedding screening within care planning and leveraging trusted relationships. Survey findings showed that most professionals acknowledged the importance of FI screening, especially during initial assessments and routine care. However, only 33% reported routinely screening for FI. Participants supported short, compassionate screening tools and highlighted the need for training, validated tools, and referral pathways.

**Discussion:**

Findings highlight the urgent need to normalise FI screening as a routine aspect of mental healthcare. Both professionals and peer workers highlighted that FI impacts mental and physical health and recovery. Flexible tools and relationally delivered screening, especially by peer workers, nurses, and community teams, were seen as most acceptable.

## Key Points

- Food insecurity is common among adults with severe mental illness but is not routinely screened for in mental health services.
- While most professionals recognised the importance of screening, only a minority reported doing so routinely.
- Peer‐support workers highlighted missed opportunities for asking about food access and stressed the importance of trust, timing, and non‐stigmatising conversations.
- Barriers to implementation included time pressures, stigma, and lack of training, tools, and referral pathways.
- Food insecurity screening was seen as most acceptable when embedded into routine care planning and delivered flexibly by trusted staff.
- Co‐produced, compassionate, and practical screening approaches may help normalise food insecurity discussions and improve support for adults with severe mental illness.

## 1 Background

Food insecurity (FI) is defined as the lack of consistent access to enough safe and nutritious food to support normal growth, health, and wellbeing [1]. FI is often the result of a combination of food unavailability and lack of financial or other resources to obtain food [1, 2]. In adults with Severe Mental Illness (SMI), FI is particularly concerning due to the potential for adverse impacts on both mental and physical health, as well as on medication adherence and treatment efficacy [3].

Food insecurity in the UK is increasing year‐on‐year, with data from the Family Resources Survey showing an increase from 6% of UK households 2020/21 to 10% in 2023/24 [4]. A systematic review conducted by the authors on FI in adults with SMI revealed a scarcity of UK‐based evidence on the prevalence of FI and highlighted a lack of validated screening tools tailored to this population [5]. Across 16 studies identified from high‐and upper‐middle income countries; none were conducted in the UK, highlighting a significant gap in the literature. A recent study by the authors found that over half of adults (50.4%) living with SMI in Northern England met the US Department of Agriculture [6] criteria for FI [5]. Together, these findings highlight an urgent need for UK‐based research regarding FI among adults with SMI and its implications for mental health treatment adherence and health outcomes.

Recent evidence further highlights the substantial impact of FI on individuals with SMI. Peer‐led qualitative research with adults living with SMI found that FI was often a long‐standing and persistent issue, shaped by unemployment, rising living costs, and fuel poverty [3]. Participants described going without food, relying on low‐cost or nutritionally poor options, and struggling to access healthier foods due to financial constraints, which in turn affected both their mental and physical health. This highlights the daily struggles of managing their mental health while navigating FI. For individuals discharged from inpatient care, the ability to access regular meals becomes a critical factor in recovery, as some medications prescribed for SMI require food for optimal efficacy [7]. Earlier research, funded by the National Institute for Health and Care Research (NIHR) Research for Patient Benefit programme, uncovered instances where service users had to choose between taking their medication on an empty stomach or forgoing it altogether due to their inability to secure food [3]. Such decisions not only affect medication adherence and treatment outcomes but also have broader implications for their physical and mental health, potentially escalating healthcare costs.

Mental health practitioners may not currently prioritise FI screening [8]. A recent systematic review identified limited evidence on the role of Registered Dietitians in FI screening, but no evidence of impacts on quality of life or nutritional status [9]. This omission is particularly worrying given the critical role of nutrition in medication adherence and overall health.

In conclusion, there is a critical need to understand and address FI among individuals with SMI in the UK. The broader impacts of FI, including its detrimental effects on mental health, treatment efficacy, and overall wellbeing, necessitate the development and implementation of effective screening tools and interventions. This study aimed to better understand how FI screening could be embedded into routine mental healthcare; the impact it may have on healthcare; and the implications for implementing this in practice.

## 2 Methods

### 2.1 Design

A mixed method study involving semi‐structured interviews and surveys was conducted between April 2025 and June 2025 in the North East of England. A mixed‐methods design was used, whereby qualitative (interviews) and quantitative (survey) data were collected in parallel, analysed separately, and integrated at the interpretation stage through triangulation.

The study was approved by Teesside University [10] ethical committee, and the Health Research Authority (Ref: 25/HRA/0041).

### 2.2 Sample and Recruitment

Participants were purposively recruited from health and social care settings across the Tees, Esk and Wear Valleys NHS Foundation Trust (TEWV) area. Recruitment was facilitated through TEWV contacts known to the researchers.

Peer Support Workers (PSWs) with lived experience of SMI were purposively sampled because of their dual positionality as both service users (with lived experience) and employees embedded within mental health services. Eligible PSWs were adults (18 + ) with lived experience of SMI (schizophrenia, bipolar disorder, or psychosis), employed within TEWV, able to provide informed consent, and English‐speaking.

Potential interview participants were identified through PSW service leads and invited via email. The invitation included a participant information sheet outlining the study purpose, voluntary nature of participation, and confidentiality procedures. Interested individuals contacted the research team directly. No incentives were offered for survey participation beyond the voucher described below. Nobody who was approached refused to take part.

The sample adequacy was determined pragmatically due to the limited number of PSWs within the Trust and feasibility constraints. The aim was depth of experiential insight rather than saturation in the traditional sense. While full thematic saturation cannot be claimed, interviews provided sufficient informational power to address the research aim, given the specificity of the sample, narrow research focus, and high relevance of participants' lived experience.

All clinical and allied health professionals working within adult mental health services across the TEWV footprint (Durham and Darlington, Teesside, North Yorkshire, York, and Selby, including secure services) were considered eligible if they: [1] worked with adults with SMI, [2] were employed by TEWV or affiliated partner services, and [3] were able to provide informed consent.

The survey link was disseminated via Trust‐wide staff email distribution lists, internal newsletters, and team leads, who were asked to cascade the invitation to all eligible colleagues. Therefore, the survey was available to all eligible staff within the relevant services rather than a selected subsample. Participation was voluntary and no exclusion criteria were applied beyond those listed above.

### 2.3 Consent, Anonymity and Incentives

For interviews, participants received an information sheet at least 48 h prior to participation and provided written informed consent before the interview commenced. Interviews were audio‐recorded with permission. Participants received a £20 Amazon voucher in appreciation of their time. For the survey, participants accessed an online information sheet prior to participation and provided informed consent electronically by ticking a mandatory consent box before proceeding. The survey was completed anonymously; no identifying information was collected within the survey itself. At the end of the survey, participants were invited to optionally provide an email address via a separate, unlinked form if they wished to receive a £10 Amazon voucher. Email addresses were stored separately from survey responses and could not be linked to individual survey data.

### 2.4 Data Collection

#### 2.4.1 Interviews

Participants chose to take part face‐to‐face, by telephone, or via Microsoft Teams. All interviews were conducted by one researcher (ET), a female PhD candidate with experience in qualitative mental health research.

An 18‐question semi‐structured interview guide was developed collaboratively by the research team and informed by existing literature on FI and SMI. The guide included open‐ended questions with prompts to allow flexibility in sequencing and depth of exploration. While core questions were asked consistently across interviews, follow‐up questions and probing were responsive to participants' answers; therefore, the interviews were semi‐structured rather than structured. The full interview schedule is provided in Supporting File 1.

Interviews lasted approximately 60 min, were audio‐recorded, transcribed verbatim, anonymised, and stored securely on a password‐protected platform accessible only to the research team.

#### 2.4.2 Survey

Surveys were administered online via JISC Surveys and included Likert‐scale, multiple‐choice, and open‐ended questions. The survey instrument was developed and informed by three PPIE members with lived experience of SMI to ensure clarity and sensitivity. The full survey instrument is provided in Supportin File 2.

The survey took approximately 15 min to complete and was open between April and June 2025.

### 2.5 Analysis

#### 2.5.1 Qualitative Analysis

Interview transcripts were analysed using reflexive thematic analysis [11]. An inductive approach was adopted, meaning codes were generated from the data rather than imposed beforehand. Analysis followed Braun and Clarke's six‐phase framework: familiarisation, coding, theme generation, theme review, theme definition, and write‐up.

Coding was conducted in NVivo 14 by ET. An explicit coding tree was developed iteratively, beginning with initial semantic codes, which were then grouped into candidate themes through constant comparison. Themes were reviewed against the dataset to ensure coherence and distinctiveness.

Although ET conducted primary coding, analytic credibility was strengthened through regular reflexive discussions with ELG and JS, during which developing codes, theme boundaries, and interpretations were reviewed and refined. A sample of transcripts (25%) was independently reviewed by a second researcher (JS) to enhance rigour and challenge interpretive assumptions.

#### 2.5.2 Survey Analysis

Quantitative survey data were analysed using descriptive statistics (frequencies, percentages, means) in Microsoft Excel.

Open‐ended survey responses were analysed using qualitative content analysis [12]. Content analysis was primarily descriptive, involving categorisation of recurrent concepts. These categories were subsequently compared with themes generated from the interview data during triangulation.

### 2.6 Mixed‐Methods Integration

Following separate analyses of survey and interview data, findings were compared using a convergence coding matrix to identify areas of agreement, complementarity, or divergence. This process enabled the development of integrated themes presented in the Results section. Integration did not occur at the data collection stage but during analytical interpretation.

### 2.7 The Research Team and Reflexivity

The academic research team were all female, consisting of one research associate (ET), and two project leads (ELG, JS), one of whom has a PhD (ELG). ET is a PhD candidate and research associate with experience in mental health research and qualitative methods. ELG is a research professor with expertise in diet‐related conditions and qualitative methods. JS is a Senior Clinical Academic Dietitian with 28 years' clinical experience in mental health settings.

The research team acknowledge that their professional backgrounds in nutrition and mental health may have shaped the framing of FI as a health issue. Reflexive discussions were held throughout analysis to consider how positionality, assumptions about social determinants of health, and power dynamics between researcher and participant may have influenced data generation and interpretation.

### 2.8 PPIE

Three PPIE members with lived experience of SMI contributed to this study.

PPIE involvement was focused specifically on the development of the semi‐structured interview topic guide. As the interviews were to be conducted with PSWs with lived experience of SMI, PPIE members were consulted to ensure that the questions were appropriate, accessible, and sensitive. They were invited to review the draft interview schedule and comment on whether the questions were understandable and easy to read, whether the wording was clear and non‐stigmatising, whether the number of questions felt appropriate, whether any important topics were missing, and whether the order and flow of the questions felt logical and comfortable.

Based on this feedback, refinements were made to simplify language, improve clarity, and adjust the sequencing of questions. Their input helped ensure that the interview schedule was acceptable and appropriately framed for individuals with lived experience of SMI.

## 3 Results

### 3.1 Participant Characteristics

#### 3.1.1 Surveys

A broad range of professionals responded (*n* = 40), including psychiatrists (*n* = 14), dieticians (*n* = 7), psychologists (*n* = 3), nurses (*n* = 3), social workers (*n* = 2), and others (*n* = 11). “Other” responses included social care coordinators (*n* = 2), psychotherapists (*n* = 2), occupational therapist (*n* = 1), physiotherapist (*n* = 1), junior doctor (*n* = 1), physical health practitioner (*n* = 1), peer practitioner (*n* = 1), trainee doctor (*n* = 1), and higher assistant psychologist (*n* = 1). All participants had 1–5+ years in mental health services: Less than 1 year (*n* = 2), 1‐3 years (*n* = 15), 3‐5 years (*n* = 7), 5+ years (*n* = 16). Responses were fairly even across regions: Durham & Darlington (*n* = 13), Teesside (*n* = 7), Secure Services (*n* = 4), and North Yorkshire, York, and Selby (*n* = 16) (Table 1).

**Table 1:** Survey Summary Table.

Domain	Quantitative findings (*n*, %)	Qualitative themes from open‐ended responses (Content analysis)	Example quote
1. Demographics & Professional Background	Roles: Psychiatrists (14), Dieticians (7), Psychologists (3), Nurses (3), Social Workers (2), Other (11). Experience: < 1 year (2), 1–3 years (15), 3–5 years (7), 5+ years (16). Regions: Durham & Darlington (13), Teesside (7), Secure (4), NYYS (16).	—	—
2. Awareness & Current Practice	63% aware of FI in SMI; 33% screen routinely.	Screening largely informal and verbal; limited use of standardised tools; reliance on clinical judgement or observation.	“If I am concerned… I will ask their permission to visually check their food supplies.”
	Few use standardised tools (4%); some used other methods (13%).		
3. Importance of Screening	55% very important; 35% important; 8% neutral; 3% not important.	Vulnerability of SMI population; link to basic needs and health; holistic care; barriers to disclosure; feasibility concerns.	“SMI patients are among the most affected by food insecurity.”
4. When to Screen	Initial assessment (83%); routine check‐ups (73%); follow‐ups (68%).	Should be ongoing, not one‐off; ideally after rapport established; contextual sensitivity needed.	“Not necessarily a one‐off question, rather regular assessment.”
5. Where to Screen	Primary care (90%); mental health clinics (75%); social care (75%); secondary care (73%).	Screening viewed as shared responsibility across services; trust and comfort influence appropriateness.	“I believe any setting is appropriate for screening for food insecurity.”
6. Number of Questions	Preferred: 2 (38%) or 6 (55%). 1 (8%); 10 (10%); 18 (0%).	Preference for short but flexible tools; avoid overload; allow exploration where needed.	“3–5 questions… not overload but allow for further exploration.”
7. Question Comfort	Most comfortable: skipping meals (85%); food not lasting (73%); food running out (70%). Least comfortable: money‐specific wording (60–63%).	Some questions may require simplification or adaptation (e.g., for learning disability services); sensitivity in wording important.	“All appropriate however my service is LD and I don't feel some of these are very LD friendly phrasing.”
8. Barriers to Implementation	Time (60%); patient discomfort (57%); lack of training (53%); role confusion (9%); pathway gaps (9%).	Structural barriers; training gaps; lack of referral pathways; fear of opening issues without solutions.	“Staff need to be well trained in where to signpost…”
9. Resources Needed	Training (83%); validated tools (75%); referral pathways (75%); guidelines (35%).	Need for institutional frameworks, practical guidance, and sustainable support systems.	“Not enough information or guidance on food insecurity.”
10. Impact & Worthwhileness	95% reported screening is worthwhile.	Strong alignment with holistic, biopsychosocial and recovery‐oriented care models; minor feasibility concerns.	“Food is a basic human need… it makes sense that this will have negative impact on their mental health.”

#### 3.1.2 Interviews

A total of four PSW's from TEWV participated in the interviews. There were a total of two males and two females. Nobody who was approached refused to take part. The locations of the participants included; Durham, Easington, Ferryhill, and Spennymoor (Table 2).

**Table 2:** Matrix display of interview themes across participants (Peer Support Workers).

Theme/subtheme	P1	P2	P3	P4
**Lived experience of food insecurity (FI)**				
Historical access	✓	✓	✓	✓
Current challenges	✗	✗	✓	✓
**Impacts of FI**				
Emotional impacts (guilt/shame)	✓	✓	✓	✓
Health impacts (mental & physical)	✓	✓	✓	✓
**Healthcare engagement**				
Previously asked about FI in healthcare	✗	✗	✗	✗
**Screening preferences**				
Timing preferences (when to ask)	Check‐ups	Early & repeated	If symptoms arise	At reviews/checks
Preferred professional (who should ask)	GP, Peer	Any	Nurse preferred	Nurse, OT, CPN
Preferred format (method of screening)	Face‐to‐face	In‐person/depends	Face‐to‐face	Mixed/flexible
Question design (length and language)	6–8, simple	6 Q, simple	Up to 10, balance	Core + optional
**Implementation factors**				
Barriers to implementation (time/comfort)	Short appts	Stigma, crises	Professional fear	Doctor admin burden
**Proposed solutions**				
Peer support role	✓	✓	Maybe/indirect	✓
Community resources	✓	✓	✓	✓

*Note:* Footnote: ✓ = explicitly discussed; ✗ = not discussed/raised in interview.

Abbreviations: CPN = Community Psychiatric Nurse, GP = General Practitioner, OT = Occupational Therapist.

### 3.2 Triangulated Findings

To better understand the opportunities and challenges for embedding FI screening within routine mental healthcare, we undertook a triangulated analysis of survey data and semi‐structured interviews. This approach allowed us to combine broad insights from the survey with detailed understanding from the interviews, helping to identify shared themes, differences, and areas of agreement.

#### 3.2.1 Awareness and Recognition of FI

Survey data indicated that 63% of professionals were aware that FI affected their patients with SMI. However, only 33% reported screening for FI routinely, with 43% stating they did not screen at all, and 25% only doing so sometimes. In the free‐text, participants often acknowledged that *“SMI patients are among the most affected by food insecurity, and many do not ask for help unless it's offered”* and that professionals may be *“the only person a patient sees, so we have a duty to screen”*.

In contrast, all PSW's described never having been asked about their food access during healthcare interactions, even in the context of visible weight loss or disordered eating. As one participant put it: *“Nobody in cancer care or anyone's asked ever really… they ask about sleep and exercise… but yeah, not really food”* (P4). Another recalled: *“When I was skinny back in the day, they did say, am I eating all right? That's really the closest I've ever been to being asked that question”* (P1).

This highlights a clear gap between awareness and action; although professionals recognise food insecurity as a problem, this understanding rarely translates into practice. As the qualitative findings suggest, this also represents a missed opportunity for early support.

#### 3.2.2 Impacts of FI on Mental and Physical Health

Both datasets revealed strong alignment in recognising the adverse consequences of FI on mental and physical health. Over half of professionals rated screening as “very important,” with free‐text responses emphasising how FI can worsen functioning, delay recovery, and that *“undoubtedly community plans for a patient will fail if they cannot sustain their basic needs”*. Others noted how FI can be misinterpreted: *“occasionally food insecurity can present as poor appetite in patients with low mood which can be seen as a mental health problem rather than a social problem and ends up being treated the wrong way.”*

Similarly, PSW's described the physical exhaustion, low mood, and shame associated with inadequate nutrition. One participant reflected on the guilt tied to caregiving: *“Lucy's 7, she has unfortunately had a lot more pizza and chicken nuggets chucked on her… I do feel guilty”* (P4). Others highlighted the mental and physical toll: *“I had obsessions with pistachio… I realised I was not having no vitamins… my mental health proper dipped”* (P1). Emotional dimensions such as guilt, particularly in relation to caregiving, emerged in the interviews and extended beyond the biomedical framing often found in the survey responses; *“When I can't get the food I want… it makes us feel quite down, quite frustrated… it takes more effort”* (P3). This highlights the varied ways FI impacts health, identity, and recovery, which may not be fully captured through standard screening practices.

#### 3.2.3 Timing and Context for Screening

Most professionals endorsed screening during initial assessments (83%), routine check‐ups (73%), and follow‐ups (68%), with a smaller proportion suggesting crisis points or CPA reviews. Free‐text responses expanded on this, with many suggesting that FI screening should be *“an ongoing issue”, “screened regularly as circumstances change”*, or even integrated into *“every contact opportunity”*. Staff stressed the importance of trust: *“Some people may not wish to disclose all information when they first start working with a service. It would be good for patients to be asked at regular intervals… when they have developed therapeutic relationships.”*

PSW's, however, emphasised the importance of relational trust and timing: they felt FI should be raised after rapport is established or during periods of stability rather than acute crisis. *“I've worked in the crisis team before and I wouldn't be asking anyone there how their diet was really, if they're in the middle of having a mental health crises… it depends on a lot of things”* (P2).

While survey respondents acknowledged contextual barriers (e.g., time and setting), qualitative accounts provided richer insights into how power dynamics and personal readiness influence willingness to disclose.

#### 3.2.4 Preferred Personnel and Methods of Screening

Professionals overwhelmingly agreed that FI screening is the responsibility of all frontline staff, but free‐text responses frequently stressed primary care, social care, and “every setting” as important points of enquiry: *“I believe everyone at all clinics can help identify food insecurity. The stigma needs to be removed and people should feel able to express when they are struggling.”*

However, PSW's consistently favoured being approached by practitioners with more time and continuity, such as nurses, occupational therapists, community psychiatric nurses (CPNs), or PSW's. General practitioners (GPs) were viewed as less suitable due to time pressures and administrative burdens. *“[Doctors] only have 10 min per person… that is wild… it's stupidly small”* (P1). They also stressed the relational quality of the interaction: *“I just think doctors tend to be very overwhelmed with admin… the nurses seem to take a bit more time and actually have a discussion with you.”* (P4).

Both datasets supported the idea that screening should not be overly formalised or delegated solely to one professional group, but qualitative data highlighted that who asks and how it is asked can significantly influence disclosure and comfort.

#### 3.2.5 Design and Delivery of Screening Questions

Survey results showed a preference for brief screening tools, with most respondents favouring 2–6 questions. Concerns were raised about overly complex phrasing or items that might be stigmatising or conflate FI with symptoms of mental illness.

In contrast, PSW's expressed a willingness to engage with longer and more open‐ended screening tools, up to 10 questions, provided the approach was compassionate and tailored. *“Too little and I wouldn't feel cared about, too many and I'd get overwhelmed… maybe around 10 questions”* (P3). Participant 4 added: *“Maybe 5 to 10 core questions and then have some optional ones… if you feel like the person you're talking [to] is not engaging… have the optional ones for data collection”* (P4).

Participants advocated for simple, relatable language and flexibility in format (e.g., face‐to‐face, telephone, or form‐based). One noted: *“Not necessarily, can you afford to eat? It's more kind of do you feel like you're able to have access to and prepare and whatever like foods that you think are nutritious”* (P4). These preferences suggest divergence in perspectives around brevity and burden, highlighting the importance of being informed by lived experience to ensure tools are acceptable, effective, and context‐appropriate.

Both datasets emphasised the need for clarity, compassion, and flexibility. Staff flagged wording pitfalls (e.g., *“balanced meals”* may confuse patients), while PSW's called for non‐stigmatising phrasing.

#### 3.2.6 Barriers to Implementation

Barriers reported by professionals included time constraints (60%), patient discomfort (57%), and lack of training (53%). Free‐text responses expanded these: *“Staff need to be well trained in where to signpost… often signposting to another organisation where they have to explain their story again can be off putting.”* Another respondent warned that there could be a *“lack of perceived usefulness if there is no follow‐up, intervention or signposting”*

These themes strongly resonate with qualitative data. For example, stigma was described as a significant obstacle: *“If somebody says, do you have enough food? The assumption is, you're not doing very well… I suppose it's just eliminating those stigmas around food insecurity, you know, around food poverty”* (P3). Others highlighted professional hesitancy: *“GP's are just as much human… I won't ask that question just in case, it might upset somebody”* (P3). Participants also reflected on situational inappropriateness: *“I wouldn't be asking anyone [in crisis] how their diet was really… it depends on a lot of things”* (P2).

Both datasets pointed to the necessity of training and cultural shifts within healthcare environments to normalise discussions around FI.

#### 3.2.7 Enablers and Opportunities for Integration

Professionals identified access to training (83%), validated tools (75%), and referral pathways (75%) as critical enablers. Free‐text suggestions emphasised sustainability: *“support around budgeting on a low income, finding low cost food options, including apps that help share places where low cost food can be sourced would be beneficial.”*

PSW's echoed the need for better signposting but also emphasised the role of community knowledge, peer support, and compassionate delivery. For example: *“In Chester St. there's a Refuse Cafe… they often do pay‐as‐you‐feel thing… but a lot of the time people don't know about this stuff in fairness”* (P4). Another stressed the importance of visibility: *“If they had pamphlets… if you're too shy to ask, you can see it… the libraries have a lot of stuff”* (P1).

PSW's also highlighted their unique role: *“Me as a peer worker is in a really good position to be talking to people about this… I can [also] see social workers being very up on this”* (P4). Additionally, they highlighted interventions such as “pay‐as‐you‐feel” cafes and suggested making resources visible in healthcare settings to reduce shame associated with disclosure.

Screening was seen not just as a clinical task but as a way to build trust and validate people's experiences. These insights highlight that practical support (like tools, training, and referral pathways) need to be paired with relational ones (such as trust, peer involvement, and community understanding) to make FI screening truly effective.

#### 3.2.8 Summary

Overall, the triangulated analysis showed strong agreement on the importance of FI screening and the barriers that make it difficult to implement. Both professionals and PSW's highlighted the need for flexibility, trust, and holistic care, though PSW's placed more emphasis on emotional and relational factors. Differences emerged around the preferred length, wording, and delivery of screening tools, highlighting the importance of co‐production in their design. Together, these perspectives point to the need for a more holistic model of FI screening that combines relational understanding, organisational support, and lived experience.

## 4 Discussion

This study provides important insights into how FI screening can be embedded into routine mental healthcare for adults with SMI. The findings reinforce and build on existing evidence that food insecurity is both widespread and deeply impactful in this population yet remains largely overlooked within the healthcare system [5, 13]. The combined perspectives of healthcare professionals and PSW's highlight a significant gap between awareness and action; while most clinicians acknowledged the importance of FI screening, few implemented it in practice. This gap highlights a significant divide between professional recognition and practical implementation, a theme echoed in broader research on social determinants of health in mental healthcare [14, 15].

### 4.1 FI as a Determinant of Health and Recovery

FI is increasingly recognised as a structural determinant of health, directly shaping both physical and mental outcomes [2]. For adults with SMI, the impacts are particularly serious due to the complex links between nutrition, medication adherence, and the management of psychiatric symptoms [7, 16]. Participants in this study described FI as intensifying fatigue, depression, shame, and impaired caregiving capacity, findings consistent with prior qualitative work documenting how FI compounds psychosocial stressors in SMI [3]. Moreover, the narratives highlight how FI may be misinterpreted as a psychiatric symptom rather than a social need, risking inappropriate treatment responses. This supports arguments for embedding FI screening within holistic models of mental healthcare that acknowledge both biomedical and social determinants of recovery [17].

### 4.2 Acceptability and Relational Dynamics of Screening

A central theme emerging from this study is that the acceptability of FI screening depends heavily on relational dynamics, trust, and timing. While professionals often endorsed structured, brief screening during initial assessments, PSW's emphasised that disclosure was most likely after rapport had been established and within ongoing, trusted relationships. This aligns with evidence that people with SMI may be reluctant to disclose sensitive social needs due to stigma and perceived judgment [18]. The importance of relational trust mirrors broader findings in patient‐centered care, where meaningful disclosure of psychosocial issues is contingent on continuity, compassion, and safe therapeutic environments [19].

Differences in views about screening length were telling; professionals favoured shorter tools due to time constraints, while PSW's were open to longer conversations when approached with empathy. This contrast reflects a broader challenge in healthcare research, finding the balance between efficiency and person‐centered care [20]. These results emphasise the value of being informed by lived experience in designing screening tools that are both practical for staff and acceptable to those being screened.

### 4.3 Barriers and Enablers to Implementation

Barriers to implementation identified in this study, such as time pressures, stigma, and lack of training, are consistent with barriers reported for the integration of other social risk screening initiatives in healthcare, such as housing or financial insecurity [21]. The persistence of these obstacles highlights the need for systemic interventions, including workforce development, integration of validated tools, and the creation of referral pathways.

In contrast, enablers identified, such as PSW's, community knowledge, and relationally sensitive delivery, echo growing recognition of the value of peer‐led interventions in addressing social needs [21, 22]. PSW's may be uniquely positioned to raise sensitive issues such as FI due to their lived experience and ability to model trust, which may reduce disclosure stigma. This suggests that embedding FI screening within multidisciplinary care, particularly with peer and nursing staff, could enhance acceptability and uptake.

### 4.4 Implications for Health Policy and Practice

The findings of this study highlight several implications for health policy and clinical practice. First, routine FI screening should be normalised within mental healthcare pathways, consistent with national and international priorities to address the social determinants of health [23]. However, for screening to be meaningful, it must be accompanied by clear and actionable referral pathways. Without such support, as participants cautioned, screening risks being perceived as tokenistic or even burdensome, potentially undermining trust in healthcare interactions.

Second, tackling cultural barriers such as stigma is essential. Professionals expressed hesitancy in raising FI due to fears of causing discomfort, while PSW's highlighted the shame often associated with disclosure. Training and awareness‐raising initiatives that frame FI as a legitimate health concern rather than an individual failing could reduce these barriers and enable more open dialogue. Embedding FI screening into existing care planning processes may also support normalisation, ensuring that it becomes a routine part of holistic care rather than an optional or secondary task.

Third, co‐production emerged as a vital principle for developing effective interventions. Involving people with lived experience of SMI in the design of FI screening tools ensures that language, format, and delivery are acceptable, relevant, and sensitive to service users' needs. Co‐production also helps to address the structural gaps and cultural silences that have long made FI an overlooked issue within mental healthcare. PSW's, in particular, may play a critical role in facilitating these conversations and linking individuals to appropriate community resources, given their lived experience and trusted relational position.

## 5 Study Limitations

Several limitations should be acknowledged. First, the small number of PSW's interviews limits generalisability; however, these accounts provide in‐depth insights consistent with broader literature [3, 5]. Second, the study was geographically constrained to a single NHS Trust in the North East of England, which may not reflect practices in other regions. Third, survey participation may be biased toward professionals with a prior interest in social determinants of health. Finally, the study did not capture the perspectives of service users disengaged from care or experiencing the most severe FI, groups who may face additional barriers to disclosure.

Despite these limitations, the study makes an important contribution by triangulating professional and lived experience perspectives and identifying practical, relational, and systemic strategies to embed FI screening in mental health services.

## 6 Conclusion

This study demonstrates that FI is a critical yet under‐addressed determinant of health for adults with SMI, with substantial impacts on recovery, wellbeing, and treatment adherence. While healthcare professionals acknowledge its importance, routine screening remains rare due to structural, cultural, and practical barriers. Findings suggest that effective FI screening must be co‐produced, compassionate, and integrated into trusted therapeutic relationships. Embedding screening across mental health services, supported by training, referral pathways, and peer involvement, offers a feasible and acceptable pathway toward addressing FI in this vulnerable population. Normalising FI screening within healthcare is a crucial step toward reducing health inequalities and improving outcomes for people with SMI.

## Author Contributions

**Emma Tuschick:** methodology, validation, formal analysis, investigation, writing – original draft, writing – review and editing, visualization. **Emma Giles:** conceptualization, methodology, investigation, validation, writing – review and editing, supervision, funding acquisition. **Jo Smith:** conceptualization, methodology, investigation, validation, writing – review and editing, supervision, funding acquisition.

## Conflicts of Interest

The authors declare no conflicts of interest.

## Supporting information

Supporting File 1

Supporting File 2

## Data Availability

The data that support the findings of this study are available from the corresponding author upon reasonable request.
