# Supplementary material for: A Mixed‐Methods Study Exploring How Food Insecurity Screening can be Embedded in Routine Mental Health Care for Adults With Severe Mental Illness
Source: J Hum Nutr Diet. 2026 Apr 27;39:e70257. doi: 10.1111/jhn.70257 (PMC13111974; doi:10.1111/jhn.70257)
Supplement: Supplementary file 1 — Supporting File 1 [file JHN-39-0-s002.docx]

**Supplementary File 1: Interview Schedule**

**INTERVIEW SCHEDULE**

| **OPENING** |
| --- |

- Thank you for agreeing to participate in this chat.
- During this chat we will talk about food insecurity, your experience with food, checking for food problems, how to best ask about food problems, and the support you feel you may need.
- There are no right or wrong answers to any of our questions, we are interested in your own experiences and thoughts.
- The chat should not take any longer than 60 minutes, but this depends on how much information you would like to share.
- With your permission, I would like to audio record the chat because I don’t want to miss any of your comments. All responses will be kept confidential and anonymous. This means that your name and personal information will not be included. No-one will know it is you who has taken part. We will ensure that any information we include in any study output does not identify you.
- You may decline to answer any question or stop the chat at any time and for any reason.
- If you want a break throughout, please let me know and I will pause the recording.
- Are there any questions that I can answer for you before we start?

*Researcher to turn on Dictaphone*

**Firstly, before we begin, please could I ask that you consent to taking part in this study and for it to be recorded using a Dictaphone?**

**Introduction**

1. Please tell me more about yourself (Prompts: family/friends, interests, hobbies etc.)

**Section 1: Your Experience with Food**

Food insecurity is the condition of not having access to sufficient food, or food of an adequate quality, to meet one's basic needs. Can you tell me how easy or hard it is for you to get enough food?
(For example: Do you have enough food to eat every day?)

How does having enough or not enough food make you feel?
(For example: Does it make you feel better or worse?)

**Section 2: Your Health and Food**

When you see your doctor or nurse or other healthcare professional, have they ever asked you about your access to food?
(For example: Do they ask if you have enough to eat?)

Do you think not having enough food affects your health? How?

**Section 3: Checking for Food Problems (Screening)**

Do you think doctors and nurses or other healthcare professionals should ask about whether you can access food? Why or why not?

When do you think is a good time for them to ask about food?
(For example: When you first visit them? Or at every appointment?)

Who would be best to screen you for this and why?

(for example, doctors, nurses, other healthcare professionals)

How would you prefer this to be done?

(for example face-to-face, filling out a form, over the telephone)

How long would you want to spend talking about it?

(for example, would you want it to be over quickly)

Would you be happy with one question or would you be OK with doing a screening tool?

(go into detail about screening tools)

Is it just NHS settings you think this should be done, or can you think of any other acceptable places?

(for example in the community)

**Section 4: Best Way to Ask About Food**

What kind of questions do you think doctors should ask about food?
(For example: Should they ask simple questions like “Do you have enough food to eat?”)

How can doctors ask these questions in a way that makes you feel comfortable?
(For example: Should they ask in private? Or in a nice way?)

**Section 5: Challenges and Support**

What could make it hard for doctors to ask about food?
(For example: Are there things that might stop them from asking?)

How do you think doctors can help people who don’t have enough food?
(For example: Should they tell them where to get help?)

Can you see any problems to asking about food insecurity or screening for this?

**Closing Remarks** Thank you so much for your time! Do you have any last thoughts you’d like to share about food and health?
