# Supplementary material for: A Mixed‐Methods Study Exploring How Food Insecurity Screening can be Embedded in Routine Mental Health Care for Adults With Severe Mental Illness
Source: J Hum Nutr Diet. 2026 Apr 27;39:e70257. doi: 10.1111/jhn.70257 (PMC13111974; doi:10.1111/jhn.70257)
Supplement: Supplementary file 2 — Supporting File 2 [file JHN-39-0-s001.docx]

**Supplementary File 2: Survey Questions**

#### **Section 1: Demographics**

1. What is your current role within the mental health team?
   1. Psychiatrist
   2. Psychologist
   3. Nurse
   4. Social worker
   5. Dietitian
   6. Other (please specify): ____________
2. How long have you been working in mental health services?
   1. Less than 1 year
   2. 1-3 years
   3. 3-5 years
   4. 5+ years
3. Which locality do you work in?
   1. Durham and Darlington
   2. Teesside
   3. Secure Services
   4. North Yorkshire, York, and Selby

#### **Section 2: Awareness and Current Practice**

1. Food insecurity is the condition of not having access to sufficient food, or food of an adequate quality, to meet one's basic needs. Are you aware of food insecurity issues in your patients with SMI?
   1. Yes
   2. No
   3. Not sure
2. Do you routinely screen for food insecurity in your practice?
   1. Yes
   2. No
   3. Sometimes
3. If yes, what methods do you use? (check all that apply)
   1. Verbal questioning
   2. Standardised questionnaires
   3. Other (please specify): ____________

#### **Section 3: Feasibility of Food Insecurity Screening**

1. Do you think it is important to screen patients with SMI for food insecurity?
   1. Very important
   2. Important
   3. Neutral
   4. Not important
   5. Why? - Free text answer
2. What would be the best time to screen for food insecurity? (Check all that apply)
   1. During initial assessments
   2. During routine check-ups
   3. During follow-up appointments
   4. During hospital stays
   5. Other (please specify): ____________
   6. Why? - Free text answer
3. What type of setting would be most appropriate for food insecurity screening?
   1. Primary care
   2. Secondary care
   3. Mental health clinics
   4. Social care settings
   5. Other (please specify): ____________
   6. Why? - Free text answer
4. What is your preference for the number of questions asked in the screening?
   1. 1
   2. 2
   3. 6
   4. 10
   5. 18
   6. Why? - Free text answer
5. Please select the following questions you would be comfortable asking in relation to the food insecurity screening (select all that apply)
   1. How often within the past 12 months were you worried whether your food would run out before you got money to buy more.
   2. How often within the past 12 months did the food you buy not last and you didn’t have money to get more.
   3. How often can you not afford to eat balanced meals.
   4. In the last 12 months, did you ever cut the size of your meals or skip meals because there wasn't enough money for food?
   5. In the last 12 months, did you ever eat less than you felt you should because there wasn't enough money for food?
   6. In the last 12 months, were you every hungry but didn't eat because there wasn't enough money for food?
   7. In the last 12 months, did you lose weight because there wasn't enough money for food?

#### **Section 4: Implementation and Barriers**

1. What potential barriers do you foresee in implementing food insecurity screening in mental health services?
   1. Lack of time
   2. Lack of training
   3. Patient discomfort
   4. Not relevant to practice
   5. Other (please specify): ____________
2. What support or resources would you need to successfully implement food insecurity screening in your service?
3. Training on FI issues
4. Access to validated screening tools
5. Referral pathways for patients
6. Institutional guidelines
7. Other (please specify): ____________

#### **Section 5: Conclusion**

1. How confident are you that food insecurity screening can help improve mental health outcomes in people with SMI?
2. Very confident
3. Confident
4. Neutral
5. Not confident
6. Do you think food insecurity screening in mental health services is worthwhile?
7. Yes
8. No
9. Why – free text answer

Thank you for completing our survey.

If any of the questions have impacted you and you wish to receive further support, please see;

Mental health:

<https://www.nhs.uk/nhs-services/mental-health-services/where-to-get-urgent-help-for-mental-health/>

<https://www.mind.org.uk/information-support/helplines/>

Food insecurity:

<https://www.trussell.org.uk/>

<https://stockton.gov.uk/article/9319/Food-insecurity-and-poverty>

**Thank You!**

We appreciate your time and insights in completing this survey. Your feedback is invaluable in helping us understand and address the challenges of food insecurity in mental health services.

As a token of our gratitude, we invite you to participate in a follow-up survey where you can claim your voucher. Please click the link below to proceed to the voucher survey:

**Access Voucher Survey Here**

*Participants will then be taken to a new anonymised survey which is not linked to their previous answers*

**Voucher Survey**

Thank you for participating in our research survey! To claim your e-voucher, please provide your email address below.

1. Email Address:
Please enter the email address where you would like to receive your e-voucher:

Your information will be handled in accordance with our data protection policies and used solely for the purpose of distributing the e-voucher.
